# Increasing Motor Noise Impairs Reinforcement Learning in Healthy Individuals

**DOI:** 10.1523/ENEURO.0050-18.2018

**Published:** 2018-08-13

**Authors:** Amanda S. Therrien, Daniel M. Wolpert, Amy J. Bastian

**Affiliations:** 1 Kennedy Krieger Institute, Johns Hopkins School of Medicine, Baltimore, Maryland 21205; 2 The Solomon H. Snyder Department of Neuroscience, Johns Hopkins School of Medicine, Baltimore, Maryland 21205; 3 Department of Engineering, University of Cambridge, Cambridge CB2 1PZ, United Kingdom; 4 Zuckerman Mind Brain Behavior Institute, Department of Neuroscience, Columbia University, NewYork 10027

**Keywords:** cerebellum, motor learning, motor noise, reinforcement learning, variability

## Abstract

Motor variability from exploration is crucial for reinforcement learning as it allows the nervous system to find new task solutions. However, motor variability from noise can be detrimental to learning and may underlie slowed reinforcement learning performance observed in individuals with cerebellar damage. Here we examine whether artificially increasing noise in healthy individuals slows reinforcement learning in a manner similar to that seen in patients with cerebellar damage. Participants used binary reinforcement to learn to rotate their reach angle in a series of directions. By comparing task performance between conditions with different levels of added noise, we show that adding a high level of noise—matched to a group of patients with cerebellar damage—slows learning. In additional experiments, we show that the detrimental effect of noise may lie in reinforcing incorrect behavior, rather than not reinforcing correct behavior. By comparing performance between healthy participants with added noise and a group of patients with cerebellar damage, we found that added noise does not slow the learning of the control group to the same degree observed in the patient group. Using a mechanistic model, we show that added noise in the present study matched patients’ motor noise and total learning. However, increased exploration in the control group relative to the group with cerebellar damage supports faster learning. Our results suggest that motor noise slows reinforcement learning by impairing the mapping of reward to the correct action and that this may underlie deficits induced by cerebellar damage.

## Significance Statement

Understanding the contribution of reinforcement mechanisms to human motor learning has been the subject of renewed interest. Exploration (i.e., varying one’s movement) is crucial to reinforcement learning. Yet, motor variability can arise from multiple sources, and the manner in which these influence learning remains poorly understood. Here, we show that artificially increasing motor noise variability slows reinforcement learning in a manner similar to that observed in people with cerebellar damage. Importantly, we show that the detrimental effect of noise may be the attribution of reinforcement to incorrect behavior, rather than not reinforcing correct behavior. These findings indicate that variability from noise may not be accessible to reinforcement learning mechanisms, which sheds light on the mechanism of deficit following cerebellar damage.

## Introduction

When a novice player is learning to shoot in basketball, he or she will often perform many repetitions of the task. The player’s movements will vary considerably from one repetition to the next, resulting in a mix of successful and unsuccessful shots. The motor system can use at least two forms of learning in such tasks, error-based learning to correct visual errors in the trajectory of the ball from trial to trial (Shadmehr et al., 2010) and reinforcement learning, which monitors success and failure (Haith and Krakauer, 2013). Although it may be frustrating for the player, motor variability can be crucial for both forms of learning (Kaelbling et al., 1996; Sutton and Barto, 1998; Wu et al., 2014). It allows the motor system to explore possible solutions to the task and select the ones that yield desired outcomes (Lee et al., 2012; Dam et al., 2013; Wu et al., 2014; Pekny et al., 2015; Shadmehr et al., 2016; Dhawale et al., 2017). However, an important consideration for motor learning is how well the motor system can estimate trial-to-trial changes in behavior. In addition to exploration, motor variability also reflects noise from stochastic and/or faulty neural processing (Osborne et al., 2005; Stein et al., 2005; Churchland et al., 2006; Faisal et al., 2008; Renart and Machens, 2014). While the motor system may possess an estimate of exploration variability (Charlesworth et al., 2012; Wu et al., 2014), it may not be able to precisely estimate variability from noise (Wolpert and Ghahramani, 2000; van Beers et al., 2004; van Beers, 2009; Chen et al., 2017). Thus, a high proportion of motor variability from noise may impair reinforcement learning by disrupting the mapping between an action performed and the outcome feedback received.

Our previous study showed that increased variability from motor noise may underlie slowed reinforcement learning performance in individuals with cerebellar damage. In Therrien et al., (2016) we compared the performance of a group of patients with cerebellar degeneration to a group of age-matched control participants in a reinforcement learning task that required them to learn to rotate their reach direction. While both groups were able to alter their reaches and retain the changes, the patients with cerebellar damage learned less efficiently—that is, compared with control subjects they showed a reduced learning rate and were unable to maximize reward. Based on a model of the task, it was proposed that a significantly greater proportion of the patients’ trial-to-trial variability came from motor noise, which led to a discrepancy between perceived and actual hand location This suggests that mapping between perceived hand location and reward would be more variable for this group, with some reaches rewarded when perceived to be outside the target zone and others unrewarded when perceived to be inside it (due to noise moving the hand inside or outside the target zone, respectively). This added noise was hypothesized to underlie slowed learning in the patient group relative to that of control subjects. However, it remained to be tested whether augmenting noise in the control group would disrupt performance in a manner similar to that seen in patients with cerebellar damage (Miall and Galea, 2016).

Here we examine whether adding external noise to the reaches of neurologically healthy individuals slows the learning of a new reaching movement in a reinforcement learning task. By comparing task performance between two conditions with different levels of added noise, we show that adding a small level of noise does not slow learning, but that adding a larger level—matched to a group of patients with cerebellar damage—does. To understand whether this slowed learning was indeed mediated by the added noise or an overall reduction in the reinforcement rate, we conduct a second experiment in which we clamped the reinforcement rate to match that observed in the high-noise condition. We show that reducing reinforcement for correct behavior does not slow learning, suggesting that the detrimental effect of noise is the reinforcement of incorrect reaches. Finally, by comparing performance between healthy individuals with external noise added to their movement and a group of patients with cerebellar damage, we find that the added noise did not slow learning in the control group to the same degree observed in the patient group. Using a mechanistic model, we find that added noise in our task leaves sufficient exploration variability to allow control subjects to learn faster than patients with cerebellar damage, although increased noise prevents them from learning optimally. We suggest that this may be attributed to a discrepancy between the nature of the added noise in the present study and the source of noise in patients with cerebellar damage.

## Materials and Methods

### Subjects

Eleven right-handed human participants were recruited for experiment 1 (4 males, 7 females; mean age, 25.0 ± 4.8 years). An additional 10 right-handed individuals were recruited for experiment 2 (5 males, 5 females; mean age, 25.6 ± 4.6 years). Finally, the data from a third group of 12 individuals with cerebellar degeneration (8 males, 4 females; mean age, 61.5 ± 10.0 years) is presented for comparison with the group from experiment 1. The data from the group with cerebellar damage were previously reported in the study by Therrien et al. (2016). All patients had ataxia from a degenerative condition affecting the cerebellum. Nine patients had a known genetic diagnosis, the remaining three patients had ataxia from sporadic or idiopathic cerebellar atrophy. The severity of the patients’ movement impairment was assessed using the International Cooperative Ataxia Rating Scale (ICARS; Trouillas et al., 1997). The patient group had a mean ICARS total score of 44.3 ± 18.1 of a possible 100. Further details about the characteristics of the patient group are shown in the study by Therrien et al. (2016). Group sizes were chosen to match those typically found in the field of motor learning and were not based on a priori power analysis. All study procedures were approved by the Johns Hopkins University ethical review board and all subjects gave written informed consent before participating.

**Table 1:** Results of model comparisons using Bayesian information criterion

	Three-parameter model (σ*_*m*_*, σ_e, rewarded_, σ_e, unrewarded_)	Two-parameter model (σ*_*m*_*, σ*_*e*_*)	One-parameter model (σ*_*e*_*)
Experiment 1	0	1735	392
Experiment 2	0	389	272
Cerebellar	184	0	1232

Our model comprised three parameters: the SDs of the Gaussian distributions of motor noise (σ*_m_*) and exploration following rewarded (σ*_e_*_, rewarded_) and unrewarded (σ*_e_*_, unrewarded_) trials. To examine the relative importance of each model parameter, we compared the full model to two reduced models: one where exploration variability does not depend on reward history (two-parameter model: σ*_m_* and σ*_e_*) and one that does not include motor noise (one-parameter model: σ*_e_*). Model comparisons using BIC show the three-parameter model best fit the data from experiments 1 and 2, and the two-parameter model best fit data from the group with cerebellar damage. For each experiment, we show the difference in BIC relative to the best model (i.e., the one with 0).

### Apparatus

All tasks were performed using a KINARM Exoskeleton Robot (B-KIN Technologies). We tested subjects making right arm-reaching movements in the horizontal plane below a screen that prevented them from viewing their arm. All visual feedback during the tasks was projected onto the screen surface.

### Procedure

#### Experiment 1: the effect of motor uncertainty on reinforcement-based learning

**Figure 1.**
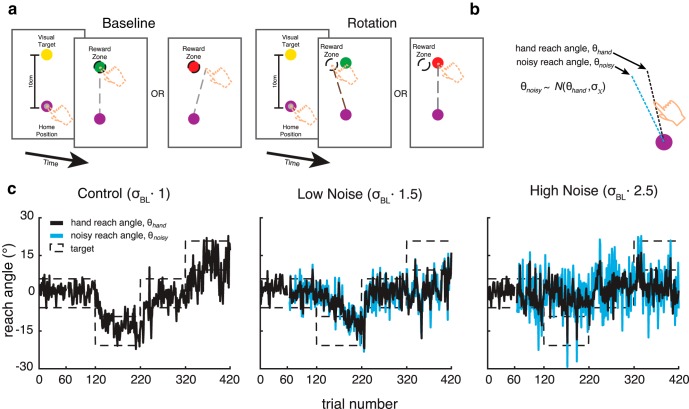
Task overview and single-subject example of noise conditions in experiment 1. ***a***, Participants were required to make 10 cm reaching movements from a home position to move their finger, through a visual target. The entire arm was hidden from view during these reaching movements. At baseline, the reward zone was centered on the visual target. In the rotation phase, the reward zone was rotated relative to the displayed target. This required participants to learn to alter their reach angle relative to the target to counter the rotation so that the finger ended in the reward zone. Participants were given a binary feedback signal informing them of reach success or failure. Reach angles that successfully fell into the reward zone were reinforced with the visual target turning green. If a reach angle fell outside of the reward zone, the visual target turned red. The reward zone encompassed reach angles between the mean of the previous 10 trials and the rotated target position. ***b***, Noise could be added to participants’ reach angles. The noise was proportional to each participant’s baseline variability, computed as the SD of the reach angles produced in the first baseline block on day 1 of the experiment, $\sigma_{bl}$. On each trial, the θ_noisy_ value was drawn randomly from a Gaussian distribution with a mean corresponding to the actual θ_hand_ and an SD such that the baseline variability would be increased by 50% (low noise) or 150% (high noise). Success or failure depended on whether the θ_noisy_ value of the finger fell within the reward zone. ***c***, Participants performed three sessions of the task on separate days, where each session corresponded to one task condition: control, low noise, or high noise. Each session began with a 60-trial baseline block with no rotation or added noise. This was followed by a second 60-trial baseline block with no rotation, but noise was added in the low-noise and high-noise conditions. Participants then performed 300 trials with closed-loop reinforcement feedback: 100 trials with the first 15º rotation; 100 trials to bring them back to zero rotation; and 100 trials with the second 15º rotation. Rotation directions and session order were counterbalanced across participants, but each participant performed the same order of rotation directions for the three experiment sessions.

Over three separate days, participants completed three sessions of a task in which they reached to a visual target with no visual feedback of the arm. They received binary feedback (success or failure) depending on whether their finger ended in a reward zone. Initially, the reward zone was centered on the visual target, but a sequence of visuomotor rotations could be applied around the home position to the (unseen) reward zone. To be successful, participants were required to learn to counteract visuomotor rotations between the location of a visual target and where they had to place their finger so as to receive reinforcement (Fig. 1*a*). In addition, noise drawn from a Gaussian distribution on each trial could be added to the finger location. Reinforcement depended on whether the noisy location was within the reward zone (Fig. 1*b*). Each session was performed with either no noise (control), or with a low-variance noise (low noise) or a high-variance noise (high noise) added (Fig. 1*c*). The three sessions were run on different days. Both rotation direction and session order were counterbalanced across participants. However, each subject performed the same order of rotation directions for all three of the experimental sessions.

Each reaching trial began with a participant’s index finger in a home position (1-cm-radius circle) located ∼40 cm in front of them. A cursor that tracked the finger position guided participants to the home position. Once the finger had been in the home position for 500 ms, the cursor disappeared and a visual target appeared on the screen (1-cm-radius circle), 10 cm distal to the home position. Participants were instructed to reach so that their index finger passed through the target. The trial ended when the index finger position exceeded a distance of 10 cm from the home position, at which time they were informed whether their movement had been successful (Therrien et al., 2016).

**Figure 2.**
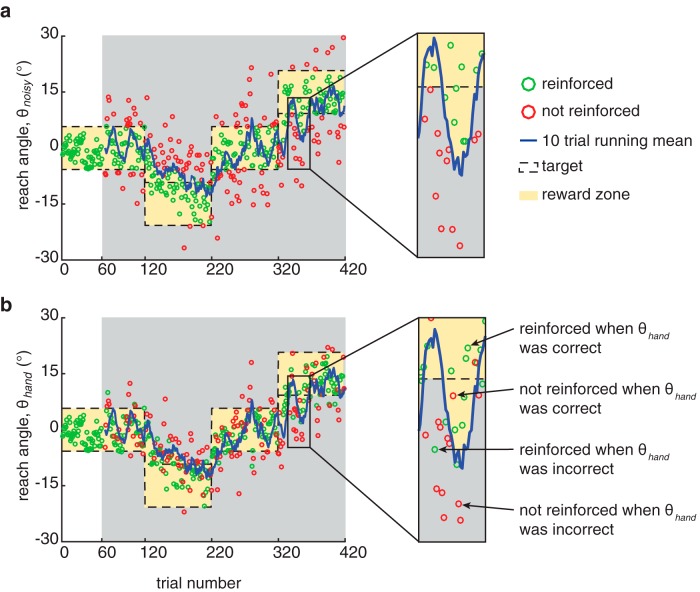
Noise influenced reinforcement. ***a***, The noisy reach angle time series from a single subject in the high-noise condition of experiment 1 plotted with the feedback received on each trial. ***b***, The actual hand reach angle time series from the same subject plotted with the feedback received on each trial. Closed-loop reinforcement feedback was based on the noisy reach angle. Thus, noise jittered reinforcement feedback, reducing participants’ ability to map reinforcement to the correct hand reach angle. As a result, participants could be reinforced when the hand reach angle was incorrect (i.e., the noisy reach angle was in the reward zone on that trial) or could be not reinforced when the hand reach angle was correct (i.e., the noisy reach angle was outside the reward zone on that trial).

In the absence of a rotation, a successful reach was one in which the finger position (with any added noise) was within 5.75º of the target (the reward zone). In the presence of a ±15º rotation, we used closed-loop reinforcement feedback. That is, a successful reach required that the finger position (with any added noise) be closer to the target than the average of the last 10 reaches (Fig. 2, reward zone). Successful reaches were reinforced with the target turning green. Incorrect reaches resulted in the target turning red. At the end of each trial, participants were instructed to relax their arm. The robot then passively returned their index finger to within 2 cm of the home position, at which time the cursor aligned with the index finger reappeared to allow participants to move their hand into the home position and begin the next trial. Participants were required to make their reaching movements within 200–600 ms after leaving the home position. To encourage this, the target turned blue or orange for movements that were too slow or too fast, respectively. These trials were then repeated until a reach was made within the time requirements.

At the beginning of each session, participants were given a 40 trial practice block with no rotation to familiarize themselves with task and desired movement speed. This was followed by two baseline blocks. The first baseline block consisted of 60 trials with no rotation or added noise (BL1). The second baseline block consisted of another 60 trials with no rotation, but noise was added in the low-noise and high-noise conditions (BL2). In the control condition, the second baseline block was identical to the first. Participants then performed 300 trials with the closed-loop reinforcement feedback: 100 trials with the first 15º rotation (either in the clockwise or counterclockwise direction; R1), 100 trials to bring them back to zero rotation, and 100 trials with the second 15º rotation (in the direction opposite to the first rotation; R2). Participants performed 420 trials per condition for a total of 1260 trials over the three experimental sessions.

In the low-noise and high-noise conditions, external noise was added to participants’ reach angles and the closed-loop reinforcement feedback given was based on the resulting noisy reach angle (θ_noisy_; Fig. 1*b*,*c*). The SD of the noise was proportional to each subject’s baseline SD, which was computed based on the reach angles produced in the first baseline block on day 1 of the experiment (60 trials). Noise was added to increase the baseline reach angle SD by 50% in the low-noise condition and 150% in the high-noise condition. The value for the high-noise condition was chosen because it approximated the difference in motor noise observed between control and groups of patients with cerebellar damage in Therrien et al. (2016).

To determine the noise that needed to be added to achieve the desired increases, we used the following calculation:

(1) $$\sqrt{\sigma_{bl}^{2}+\sigma_{x}^{2}}=\alpha \cdot \sigma_{bl}$$

Or,

(2) $$\sigma_{x}=\sqrt{\left(\alpha^{2}-1\right)}\cdot \sigma_{bl}$$

Here, α, represents the multiplier on the baseline SD in each noise condition. It was set to 1, 1.5 and 2.5 for the control, low-noise and high-noise conditions, respectively. $\sigma_{x}$, represents the SD of the noise added to the subject’s movements to achieve the desired total variability in each noise condition. This variability was added to participants’ reaches by calculating the reach angle on each trial as the index finger crossed a radius 10 cm from the home position (Fig. 1*b*, hand reach angle, θ_hand_). A new reach angle (Fig. 1*b*, noisy reach angle, θ_noisy_) was then randomly sampled from a Gaussian distribution centered on the hand reach angle and with a SD of, $\sigma_{x}$. To facilitate comparison for each subject across noise conditions and performance across subjects we used the same sequence of noise scaled by each subject's baseline variability (Thoroughman and Shadmehr, 1999; Brennan and Smith, 2015). This controlled for the possibility that any differences between the low-noise and high-noise conditions, or between subjects, was the result of differences in the particular samples drawn from the Gaussian distribution. As participants only experienced this sequence of 360 random variables twice (which only affects whether they are rewarded or not), it is unlikely that the repetition would affect performance through any form of learning.

Closed-loop reinforcement feedback was based on the noisy reach angle. Sometimes, noise did not affect the outcome of the reach, so that a correct hand reach angle was still rewarded and an incorrect one was not. Other times, noise did affect the outcome such that a correct hand reach angle would be pushed out of the reward zone by noise and not be reinforced. Conversely, an incorrect hand reach angle could be pushed into the reward zone and be reinforced. The effect of noise on reinforcement in this task is illustrated in Figure 2.

#### Experiment 2: Effect of reward rate on reinforcement-based learning

In the high-noise condition of experiment 1, participants received reinforcement on 45.8% of trials. However, only 33.8% of trials were reinforced when the hand reach angle was actually correct (i.e., the hand and noisy reach angles were both in the reward zone), and 12% were reinforced when the hand reach angle was incorrect (i.e., the hand reach angle was outside the reward zone and the noisy reach angle was in it). Thus, it was unclear whether learning in the high-noise condition of experiment 1 was slowed by the reduced reinforcement of correct reaches or the reinforcement of incorrect reaches. We tested this in experiment 2 over 2 separate days. Participants performed two sessions of the same reaching task, as in experiment 1. In one session, participants performed the same control condition as in experiment 1 (control), and in the other session the reinforcement rate was clamped at 33.8% (clamp), corresponding to the rewarded trials that were correct in the high-noise condition of experiment 1. To clamp the reinforcement, we computed the average reinforcement rate accumulated over the session and withheld reinforcement for correct movements when this average exceeded 33.8%. Importantly, all positive reinforcement feedback given was veridical (i.e., no noise was added in this experiment), but sometimes reinforcement of a correct reach was withheld to keep the average rate at the clamped value. Both rotation direction and session order were counterbalanced across participants.

### Comparison with patients with cerebellar damage

We compared the performance of participants in experiment 1 to a group of patients with cerebellar damage who had previously completed the closed-loop reinforcement learning task. In a previous experiment, subjects with cerebellar damage experienced a single 15º visuomotor rotation (this was part of a study published in Therrien et al., 2016). To match trial numbers between the two groups, we compared the final 40 trials of the second baseline block and the 100 trials of the first rotation in experiment 1 to the 40 trial baseline and the first 100 trials of the visuomotor rotation performed by the patients with cerebellar damage in the study by Therrien et al. (2016).

### Measurement and analysis

#### Behavioral analysis

We calculated the hand reach angle at the point where the finger crossed a radius 10 cm from the home position. We converted reach angle data for each subject (by appropriately flipping the signs) to correspond to a single order of rotation directions, clockwise then counterclockwise. Separate repeated-measures ANOVA was used to compare group means for reach angle and reinforcement rate. This was done using the factors condition (control, low-noise, and high-noise conditions) and block (means of the end of the first baseline block: trials 21-60; end of the second baseline block: trials 81-120; and the two rotation blocks: trials 181-220 and trials 381-420). In experiment 2, the mean reach angle was compared using repeated-measures ANOVA with factors condition (control and clamp) and the same factor of block used in experiment 1. In experiment 2, the reinforcement rate was compared across conditions using a paired-samples *t* test.

For the low-noise and high-noise conditions of experiment 1 and the control and clamp conditions of experiment 2, we performed trial-by-trial analysis of the absolute change in reach angle following rewarded and unrewarded trials (Pekny et al., 2015). For experiment 1, we parsed trials into the following four categories: rewarded when the hand reach angle was correct (rewarded-in zone); rewarded when the hand reach angle was incorrect (rewarded-out zone); unrewarded when the hand reach angle was incorrect (unrewarded-out zone); and unrewarded when the hand reach angle was correct (unrewarded-in zone). Separate repeated-measures ANOVA with factors reward (rewarded and unrewarded) and accuracy (in zone and out zone) were used to compare group means of the absolute change in reach angle for the four trial types for the low-noise and high-noise conditions. For experiment 2, trials were categorized into rewarded and unrewarded trials only because no noise was added in this task. Repeated-measures ANOVA with factors condition (control and clamp) and reward (rewarded or unrewarded) was also used to compare the absolute change in reach angle for each feedback type across control and clamp conditions.

For the comparison between experiment 1 and the sample of patients with cerebellar damage, group means for the early learning rate (slope over the first 40 rotation trials, computed using linear regression), total learning (mean of the final 40 rotation trials minus the mean of the baseline), and reinforcement rate were compared across groups using independent-samples *t* tests.

All *post hoc* analyses were performed using simple effects analysis with Bonferroni corrections on an α value of *p* < 0.05 for multiple comparisons. All data were tested for normality using the Shapiro–Wilk test. Homogeneity of variance was also examined using Mauchly’s test of sphericity and Levene’s test for the ANOVA and *t* tests, respectively. Unequal variances in ANOVA were corrected using Greenhouse–Geisser corrections to ensure that significant effects were robust to heteroscedasticity. Statistical analysis was performed using SPSS software.

#### Model analysis

We modeled subjects’ learning in experiment 1 using a simple mechanistic model that incorporates both exploration and motor noise based on the fitting procedures used in Therrien et al. (2016). The model code is available as Extended Data. The model assumes that on any trial, *t*, participants have an internal estimate of the reach angle that will lead to success, *x_t_*. When subjects make a reach based on this internal estimate, we add two sources of variability that each affect the final reach direction: exploration variability and motor noise. The key difference between these sources of variability is that it is assumed participants are fully aware of their exploration, but are unaware of their motor noise. Thus, the model assumes that there is a total variability in participants’ reaches, but they are only able to correct for a proportion of this variability.

10.1523/ENEURO.0050-18.2018.ed1 Extended Data Supplementary Computational Model Code and Example Data. Download Extended Data, ZIP file.

On each trial, exploration and motor noise are modeled as random draws from zero mean Gaussian distributions with SDs of $\sigma_{e}$ and $\sigma_{m}$, respectively. We allow for the exploration noise, $\sigma_{e}$, to take on two different values, depending on whether the last trial was rewarded or not, as variability is known to increase after a nonrewarded trial (Pekny et al., 2015). We modeled the noisy reach angle on each trial, *y_t_*, as *y_t_* = *x_t_* + *e_t_* + *m_t_*. Here, *e_t_* and *m_t_* are single draws of the exploration variability and motor noise, respectively. If a reach is not reinforced, then the internal estimate of the correct reach direction remains unchanged (*x_t + 1_* = *x_t_*). However, if the reach is reinforced, the estimate of the correct reach angle is updated by the exploration noise applied on that trial (*x_t + 1_* = *x_t_* + *e_t_*).

A particle filter was used to fit this stochastic model using the BADS optimization package (Acerbi and Ji, 2017). For each parameter setting, all particles were initialized at the average reach angle of the first baseline phase. Particles represented estimates of the perturbation such that $x_{t}^{r}$ is the rotation estimate for particle, *r*, at time, *t*. Each of the following *T* steps (corresponding to the number of trials) of the simulation involved the following:

1. computing the weight of each particle, *r*
  (3) $$w_{t}^{r}=P(y_{t}|x_{t}^{r})=N(y_{t},x_{t}^{r},\sqrt{\sigma_{m}^{2}+\sigma_{e}^{2}})$$
2. calculating an estimate of the likelihood for that data point
  (4) $$l_{t}=(1/R)\sum_{r}w_{t}^{r}\approx P(y_{t}|y_{1},\ldots ,y_{t-1})$$
3. normalizing the weights to a sum of 1 across all particles
  (5) $${\hat{w}}_{t}^{r}=w_{t}^{r}/\sum_{t}w_{t}^{r}$$
4. resampling *R* particles so that for each sample, the probability of sampling particle, *r*, corresponds to ${\hat{w}}_{t}^{r}$ and
5. If *t* < *T*, go to 1 with *t= t + 1.*

We simulated *R* = 10,000 particles for each setting of the three model parameters. For each subject, we found the parameters that maximized the log-likelihood.

To provide a measure of goodness of fit for each condition, we compared the mean reaching behavior over subjects with the mean of the predictions. By averaging in this manner, we reduce the noise components (i.e., individual draws of motor noise and exploration variability) that we do not expect the model to predict on each trial and instead measure how well the model can explain the mean behavior.

We also compared the full three-parameter model to two reduced models: one with no motor noise and one with exploration noise that does not depend on whether the last trial was rewarded or not (Therrien et al., 2016). We did not examine a model with motor noise only as such a model cannot show any learning. We used the Bayesian information criterion (BIC) to compare the models by controlling for their differing number of free parameters. To compute an overall BIC across subjects, we summed the degrees of freedom, number of trials, and log-likelihoods for each model.

Independent-samples *t* tests were used to compare experiment 1 fits to the fitted parameters for a group of patients with cerebellar damage. Paired-samples *t* tests were used to compare the fit between the control and clamp conditions of experiment 2. Model-fitting procedures were performed using software custom written in MATLAB (MathWorks).

## Results

### Experiment 1

**Figure 3.**
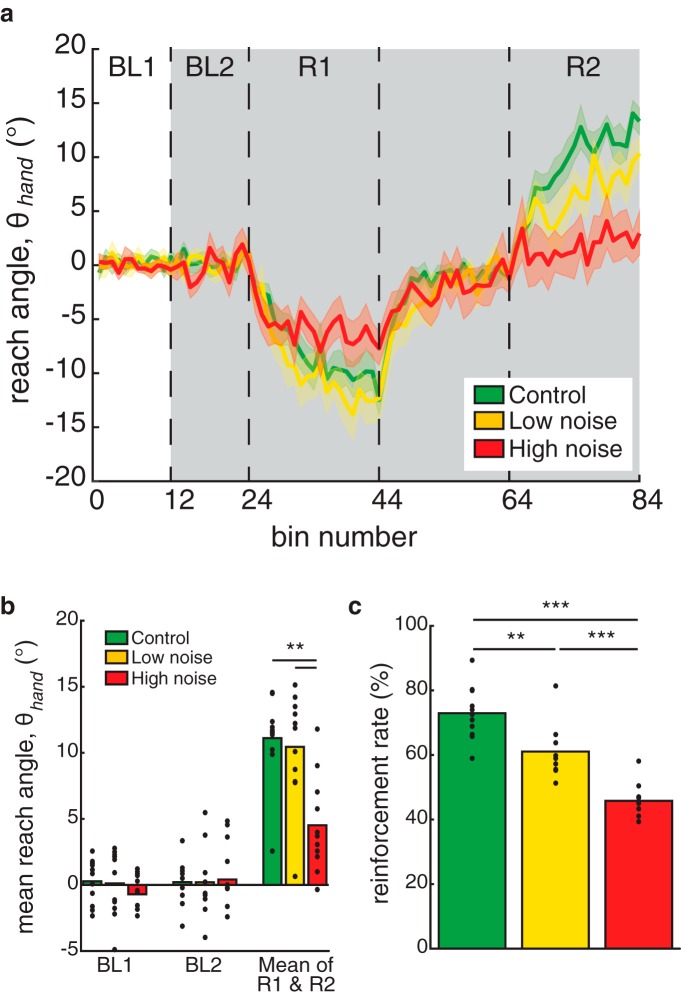
The effect of increasing motor uncertainty on reinforcement-based learning. ***a***, Reach angle (group mean ± SEM) across trials for the control, low-noise, and high-noise conditions of experiment 1. ***b***, Group mean reach angle of each condition from the first and second baseline and rotation blocks. ***c***, Group mean reinforcement rate (the percentage of reinforced trials) of each condition. Filled black circles represent individual subjects. *p < 0.05, **p < 0.01, ***p < 0.001.

The first experiment examined how adding noise affects reinforcement motor learning in healthy subjects. Figure 3*a* shows the group mean time series for all three experimental conditions in which no noise, or low or high levels of noise were added (green, yellow, and red curves, respectively). Participants’ average reaches during the baseline phase of each condition, both before and after the addition of noise, were very similar (Fig 3*b*). This suggests that simply adding noise did not impair their baseline ability to perform the task.

When subjects performed the control condition (i.e., with no noise added), they showed strong learning to the initial rotation, followed by a return to baseline, and finally strong learning of the second opposite rotation. In this condition, subjects hit a mean reach angle of 12º (in the direction countering the rotation) across the final 40 trials of each rotation phase (Fig 3*b*, green). Similar levels of adaptation (∼10º) were seen when subjects had a low noise level added (Fig 3*b*, yellow), although they had a significantly lower reward rate (61% vs 77%). However, in the high-noise condition (Fig. 3*b*, red), subjects showed significantly reduced adaptation (∼5º) and a lower reward rate (46%).

We performed a repeated-measures ANOVA for average hand reach angle in the final 40 trials of each block (Fig 3*a*, BL1, BL2, R1, and R2) and condition. This showed a significant main effect of block (*F*_(2,20)_ = 47.950, *p* < 0.001; Greenhouse–Geisser corrected, *F*_(1.248,12.480)_ = 47.950, *p* < 0.001) and condition (*F*_(2,20)_ = 10.402, *p* = 0.001) as well as a significant block by condition interaction (*F*_(4,40)_ = 12.349, *p* < 0.001; Fig. 3*b*). *Post hoc* analysis showed no significant differences between the first and second baseline blocks in any of the experimental conditions (all *p* > 0.05). However, in all three conditions, there was significant adaptation during both the first rotation block (all *p* < 0.001) and the second rotation block (control and low noise, *p* < 0.001; high noise, *p* = 0.028) compared with their respective baselines. In the rotation block, reach angle adaptation in the high-noise condition was significantly reduced compared with both the control (*p* = 0.002) and low-noise (*p* = 0.006) conditions. No significant difference was found in learning between the control and low-noise conditions (*p* = 1.00). Overall, these results suggest that participants were able to use reinforcement feedback to significantly alter their reach angle in all three experimental conditions, but this was reduced in the high-noise condition.

Figure 3*c* shows that the reinforcement rate was highest in the control condition and dropped in the low-noise and high-noise conditions (72%, 61%, and 46%, respectively; *F*_(2,30)_ = 37.405, *p* < 0.001). Note that the reinforcement rate showed a statistically significant decrease with each level of added noise (all *post hoc* tests, *p* < 0.001).

**Figure 4.**
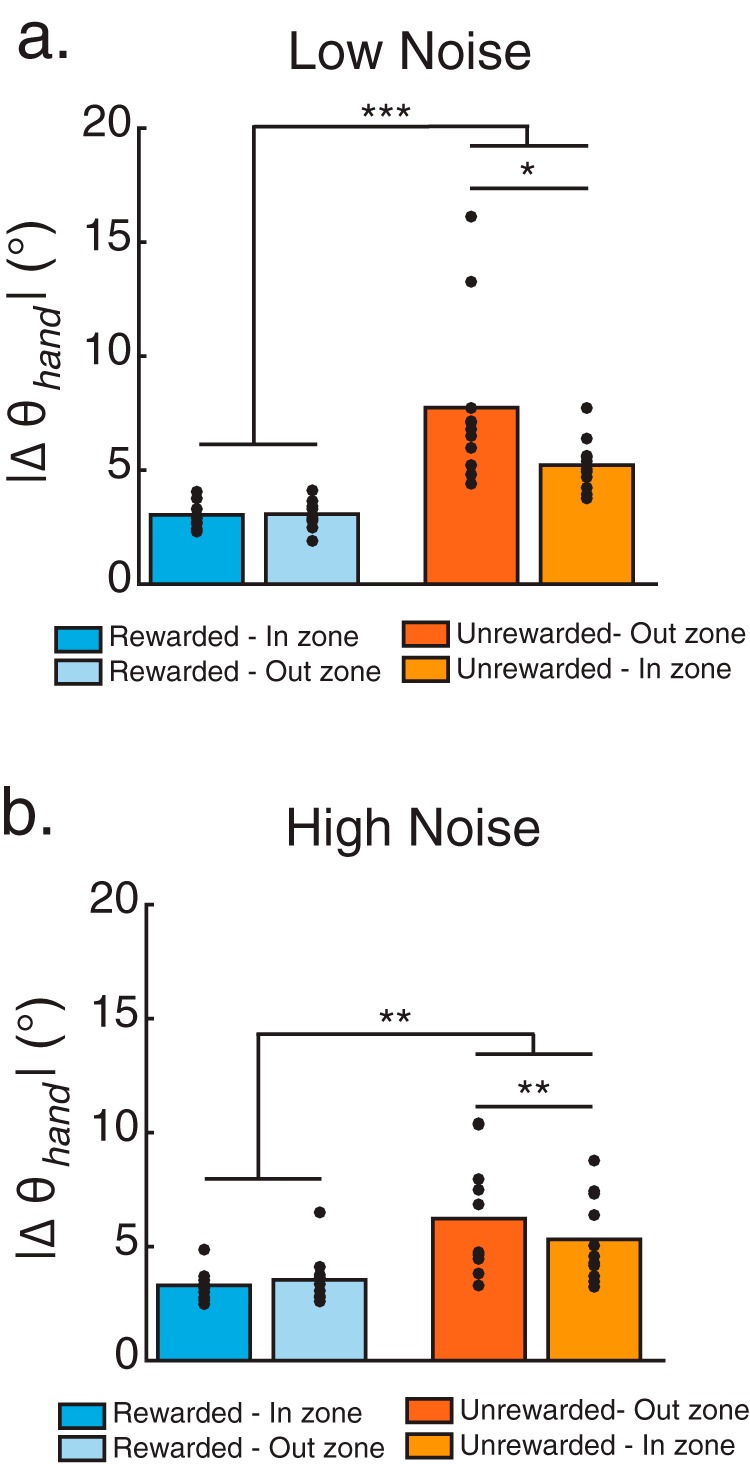
The effect of reward feedback on movement variability. The addition of noise in experiment 1 generated four potential types of outcome feedback. Participants could be rewarded when the hand reach angle was correct (Rewarded-In zone) or incorrect (Rewarded-Out zone). Conversely, reward could be withheld when the hand reach angle was incorrect (Unrewarded-Out zone) or correct (Unrewarded-In zone). ***a***, ***b***, Group means of the absolute change in reach angle after the four types of outcome feedback in the low-noise condition (***a***) and high-noise condition (***b***) of experiment 1. Filled black circles represent individuals subjects. *p < 0.05, **p < 0.01, ***p < 0.01.

To parse the influence of reinforcement feedback on subjects’ behavior in the noise conditions, we computed the absolute change in reach angle as a function of whether a trial was rewarded or not (Pekny et al., 2015) and whether the true reach was in or out of the reward zone. Figure 4 shows that subjects changed their reach angle more following unrewarded trials compared with rewarded trials, regardless of whether they were in or out of zone. The main effect of reward for the low-noise and high-noise conditions were *F*_(1,10)_ = 29.179, *p* < 0.001 and *F*_(1,10)_ = 29.179, *p* < 0.001, respectively. In the low-noise condition, there was also a main effect of reach accuracy (*F*_(1,10)_ = 9.299, *p* = 0.012). Finally, there were significant reward by accuracy interactions in both the low-noise condition (*F*_(1,10)_ = 7.006, *p* = 0.024; Fig. 4*a*) and the high-noise condition (*F*_(1,10)_ = 14.270, *p* = 0.004; Fig. 4*b*).

### Experiment 2

The second experiment examined whether slower learning in the high-noise condition of experiment 1 resulted from reinforcing errors by clamping the reinforcement rate at 33.8% (clamp) in a new group of participants. This was done to match the proportion of the trials reinforced in the high-noise task when the hand reach angle was correct. The high-noise and clamp tasks were now similar, except that the latter was not reinforced on any error trial (12% of trials in the high-noise task). To ensure that the reinforcement rate was indeed held at a fixed value throughout the clamp condition, we analyzed the group mean reinforcement rate at the following four phases of the task: the final 40 trials of the second baseline phase; the final 40 trials of the first rotation; the final 40 trials of the return to baseline; and the final 40 trials of the second rotation. Repeated-measures ANOVA showed no significant differences across the phases (*F*_(3,27)_ = 1.637, *p* = 0.204).

**Figure 5.**
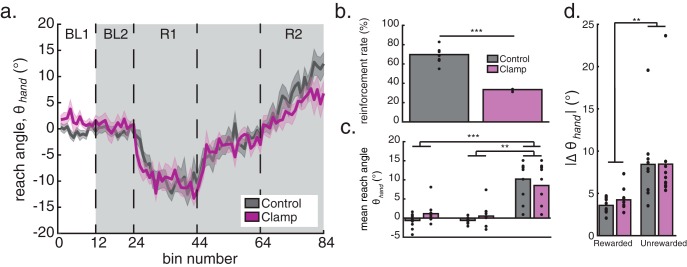
The effect of reward rate on reinforcement-based learning. ***a–d***, The effect of clamping reinforcement to match the percentage of rewarded trials in the high-noise condition of experiment 1 where the actual behavior was correct (i.e., the added noise did not push them outside of the reward zone; 33.8%). Reach angle (group mean ± SEM) across trials (***a***), group mean reinforcement rate (***b***), group mean reach angle (***c***), and group means of the absolute change in reach angle (***d***) following rewarded and unrewarded trials for the control and clamp conditions. Filled black circles represent individual subjects. **p < 0.01, ***p < 0.001.

A paired-samples *t* test showed that the difference in reinforcement rate between the control and clamp conditions was significant (*t*_(9)_ = 14.1524, *p* < 0.001; Fig. 5*b*). Figure 5*a* shows the group mean time series for the two conditions. The average reach angle during the baseline phase of the clamp condition was similar to that during the control condition, indicating that the reduction in reinforcement rate did not impair participants’ baseline ability to perform the task. We also found that the lower reinforcement rate of the clamp condition did not affect the learning of the initial rotation, return to baseline, or the learning of the second opposite rotation. Repeated-measures ANOVA showed only a significant main effect of block (*F*_(2,18)_ = 30.734, *p* < 0.001; Greenhouse–Geisser corrected: *F*_(1.286,11.572)_ = 30.734, *p* < 0.001; Fig. 5*c*). *Post hoc* analysis showed that the main effect was driven by significant differences between the two baseline blocks and the rotation block (both *p* < 0.001). There was no significant difference between the first and second baseline block (*p* = 1.00). Within both the control and clamp conditions, there was significant adaptation in both the first and second rotations (all *p* < 0.001) compared with both baseline blocks. This suggests that the reduced learning rate in the high-noise group from experiment 1 was due to the 12% of erroneous reaches that were rewarded when θ_hand_ was outside the reward zone.

As in experiment 1, we analyzed the absolute change in reach angle as a function of reinforcement feedback. Repeated-measures ANOVA showed only a significant main effect of reward (*F*_(1,9)_ = 12.490, *p* = 0.006; Fig. 5*d*). That is, participants showed a greater change in reach angle following unrewarded trials compared with rewarded trials. There was no effect of condition (*F*_(1,9)_ = 0.416, *p* = 0.535) or reward by condition interaction (*F*_(1,9)_ = 0.469, *p* = 0.511). This suggests that although participants altered their behavior following unrewarded trials more than following rewarded trials, they did not change their behavior from the control and clamp conditions.

### Comparison with patients with cerebellar damage

**Figure 6.**
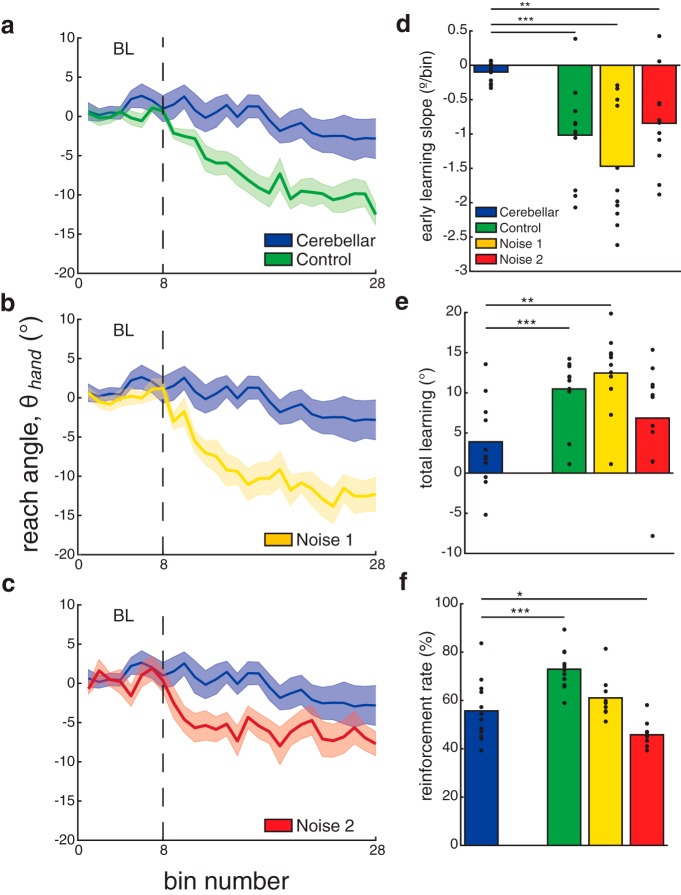
Comparison between patients with cerebellar damage and healthy individuals in experiment 1. To determine whether adding noise to reaches in healthy individuals would impair reinforcement learning similarly to what has been observed following cerebellar damage, group mean performance in experiment 1 was compared with that of a group of patients with cerebellar degeneration. ***a–c***, Reach angle (group mean ± SEM) across trials for patients with cerebellar damage plotted with the control (***a***), low-noise condition (***b***), and high-noise condition (***c***) of experiment 1. ***d–f***, Group means for the patients with cerebellar damage and the three conditions of experiment 1 of early learning slope (***d***), total learning (***e***), and reinforcement rate (***f***). Filled black circles represent individual subjects. *p < 0.05, **p < 0.01, ***p < 0.001.

We compared subjects’ learning of the first rotation in experiment 1 to the performance of a group of individuals with cerebellar degeneration who learned a single 15º rotation using the same closed-loop reinforcement feedback as part of a previous study (published in Therrien et al., 2016). Figure 6 shows the time series data comparing the performance of the group with cerebellar damage to the groups in control (Fig. 6*a*), low-noise (Fig. 6*b*), and high-noise (Fig. 6*c*) conditions of experiment 1. The group of patients with cerebellar damage was able to reach within the target zone at baseline, but were unable to reach to the new target zone in the rotation block. This differed from the control and low-noise conditions of experiment 1, where participants were able to learn to rotate their reach angles to the new target zone. Similar to the patient group, participants were unable to reach the final target zone in the high-noise condition of experiment 1. However, participants in the high-noise condition still showed a faster learning rate than patients with cerebellar damage.

Using independent-samples *t* tests, we compared the mean values of the group with cerebellar damage for early learning rate, total learning, and reinforcement rate for each condition of experiment 1 (Fig. 6*d–f*). Participants in the control condition showed significantly greater total learning (*t*_(21)_ = −3.2648, *p* = 0.004), early learning rate (*t*_(21)_ = 4.3910, *p* < 0.001), and reinforcement rate (*t*_(21)_ = −3.8324, *p* < 0.001) compared with the group of patients with cerebellar damage. Participants also showed significantly greater total learning (*t*_(21)_ = −3.9907, *p* < 0.001) and early learning rate (*t*_(21)_ = 5.3784, *p* < 0.001) in the low-noise condition compared with the patients with cerebellar damage. The difference in reinforcement rate between the low-noise condition and the group of patients with cerebellar damage was not significant (*t*_(21)_ = −1.1919, *p* = 0.247).

Finally, comparing the high-noise condition to the patients with cerebellar damage, we found that total learning was not significantly different between groups (*t*_(21)_ = −1.861, *p* = 0.249). However, there were significant differences in early learning rate (*t*_(21)_ = 3.6563, *p* = 0.002) and reinforcement rate (*t*_(21)_ *=* 2.4292, *p* = 0.024). The early learning rate was greater for participants in the high-noise condition compared with patients with cerebellar damage. However, patients with cerebellar damage showed a greater reinforcement rate than participants in the high-noise condition. Overall, these results suggest that adding noise to the reaches of neurologically healthy participants’ impaired reinforcement learning in our task, but did not completely replicate the behavior of the patient group.

### Modeling results

The objective of experiment 1 was to test the hypothesis that increased variability from motor noise would impair reinforcement learning in a similar way to patients with cerebellar degeneration. While behavioral data showed similar total learning between the high-noise and patient groups, the high-noise group showed a faster early learning rate. To determine the source of this discrepancy, we modeled subjects learning in the three conditions of experiment 1 using a simple mechanistic model of the reinforcement learning task. The reach angle executed on each trial is modeled as the sum of an internal estimate of the correct reach angle and two sources of behavioral variability, as follows: exploration variability and motor noise. The model assumes that participants have access to the variability from exploration, but do not have access to motor noise. As a result, if a reach is reinforced, the model only updates the internal estimate of the correct movement by the draw of exploration variability on that trial. When a reach is not reinforced, the internal estimate is not updated. The model has three parameters: the SDs of the Gaussian distributions that generate motor noise and exploration variability following rewarded and unrewarded trials. We fit the model to each subject’s data in each condition of experiment 1 as well as each subject’s data in each condition of experiment 2. We also fit the data for each patient with cerebellar damage. The model was fit using maximum-likelihood estimation.

**Figure 7.**
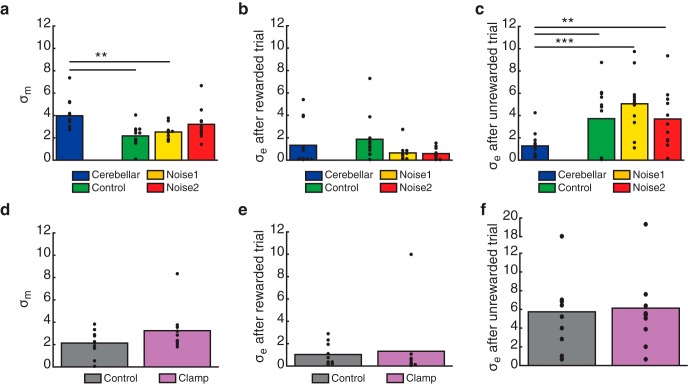
Model analysis of closed loop reinforcement learning in patients with cerebellar damage and healthy individuals in experiments 1 and 2. ***a–c***, Group means for patients with cerebellar damage and the three conditions of experiment 1 of model parameters motor noise (***a***), exploration variability following rewarded trials (***b***), and exploration variability following unrewarded trials (***c***). ***d–f***, Group means for the two conditions of experiment 2 of model parameters motor noise (***d***), exploration variability following rewarded trials (***e***), and exploration variability following unrewarded trials (***f***). Filled black circles represent individual subjects. **p < 0.01, ***p < 0.001.

Comparing mean parameter values between subjects in experiment 1 and the group of patients with cerebellar damage revealed significant differences in motor noise (Fig. 7*a*). Patients with cerebellar damage had significantly greater motor noise than participants in the control (*t*_(21)_ = 3.5707, *p* = 0.002) and low-noise (*t*_(21)_ = 3.1929, *p* = 0.004) conditions of experiment 1. The difference between patients with cerebellar damage and participants in the high-noise condition was not significant (*t*_(21)_ = 1.3225, *p* = 0.200), suggesting that this condition effectively matched the motor noise of patients with cerebellar damage. There were no differences between groups across the conditions of experiment 1 for exploration variability following rewarded trials (control condition: *t*_(21)_ = −0.6409, *p* = 0.529; low-noise condition: *t*_(21)_ = 1.0964, *p* = 0.285; high-noise condition: *t*_(21)_ = 1.2381, *p* = 0.229; Fig. 7*b*). However, patients with cerebellar damage showed significantly smaller exploration following unrewarded trials compared with participants in all three conditions of experiment 1 (control condition: *t*_(21)_ = −2.5702, *p* = 0.018; low-noise condition: *t*_(21)_ = −4.5549, *p* < 0.001; high-noise condition: *t*_(21)_ = −2.8990, *p* = 0.009; Fig. 7*c*). Thus, adding noise in experiment 1 did not reduce participants’ exploration following errors relative to the control condition. This left them greater variability from which to learn compared with patients with cerebellar damage.

There were no differences in fitted parameter values between the control and clamp conditions of experiment 2 (Fig. 7*d*: σ*_m_*, *t*_(9)_ = −1.7678, *p* = 0.111; Fig. 7*e*: σ_e_ after rewarded trial, *t*_(9)_ = −0.2686, *p* = 0.794; Fig. 7*f*: σ*_e_* after unrewarded trial, *t*_(9)_ = −0.4655, *p* = 0.653). This suggests that withholding reinforcement of correct movements did not change participants’ motor noise or exploration variability relative to the control condition.

To determine the goodness of fit of our model, we compared the mean reaching behavior over subjects in each condition of experiments 1 and 2 to the mean of the model simulations. This resulted in *R* ^2^ values of 0.95, 0.91, and 0.66 for the control, low-noise, and high-noise conditions of experiment 1. For experiment 2, *R* ^2^ values were 0.87 and 0.76 for the control and clamp conditions, respectively.

Finally, to examine the importance of each model parameter we compared the full three-parameter model to two reduced models (one with no motor noise and one with exploration variability) that did not depend on whether the previous trial was rewarded or not (Therrien et al., 2016). We did not examine a model with motor noise only because some exploration variability is needed to show learning. Model comparisons using the BIC showed that the three-parameter model best fit the data for experiments 1 and 2, while the reduced model with exploration that was independent of reward on the previous trial best fit the data for patients with cerebellar damage (Table 1).

## Discussion

We examined whether perturbing neurologically healthy individuals by adding noise to their reach endpoints would impair reinforcement learning in a manner similar to what has been observed in individuals with cerebellar damage (Therrien et al., 2016). Adding a low level of noise, to increase participants’ baseline variability by 50%, did not impair learning relative to a control condition where no noise was added. However, adding a high level of noise (to increase baseline variability by 150%) significantly impaired learning. Increasing variability affects the mapping of hand location to reward. That is, in the presence of noise it is possible for the hand to be within the reward zone yet not be rewarded or, conversely, be rewarded when outside it. To assess whether reinforcing errors could account for impaired learning with high noise, we performed an additional experiment in which we artificially reduced (clamped) the reinforcement rate to match the reinforcement corresponding to reaches where both the hand and noisy locations were in the reward zone. In contrast to the noise conditions, in this additional task participants were never rewarded when the hand location was outside the reward zone. Reducing reward yielded learning similar to that in a control condition. Together, these results suggest that the reduced learning in the high-noise condition was driven by the reinforcement of incorrect behavior, rather than not reinforcing correct behavior. Finally, comparing performance in the high-noise condition to that of a group of patients with cerebellar damage showed similar total learning between the groups, but faster early learning in the high-noise condition.

Similar to previous work (Pekny et al., 2015), we found a larger change in reach angle following unrewarded trials, suggesting that participants tend to explore more following errors than after successful movements. However, when noise was added, this exploratory behavior was modulated by whether outcome feedback matched the true hand position. That is, participants showed greater change following unrewarded trials when the hand reach angle was outside the reward zone (i.e., an appropriate withholding of reward) compared with when the hand reach angle was actually correct (i.e., a false withholding of reward). In experiment 2, the change in reach angle was also greater for the unrewarded versus rewarded trials. However, there was no difference between control and clamp conditions. This is in contrast to the results of Pekny et al. (2015), who found that clamping reinforcement at a lower level increased variability following unrewarded trials. This discrepancy may have been the result of methodological differences between the two tasks. In their study, Pekny et al. (2015) clamped the reinforcement rate during a prolonged period where no rotation perturbation was applied. Thus, many subjects would have reached a plateau in performance before experiencing the clamp. A sudden reduction in the reward rate under these conditions may have prompted subjects to change their behavior to search for a new solution to the task. In our study, however, the clamp was applied during the rotation phase. Here, subjects would naturally experience changes in the reinforcement rate as the task solution changed with each rotation. As a result, subjects in our study may have been less likely to change their behavior, relative to the control condition, on the introduction of clamp.

Adding a high level of noise to reaches of healthy participants matched the total learning of a group of patients with cerebellar damage. However, healthy participants still showed a faster early learning rate than the patient group. To describe how variability from noise influenced learning in our task, we expanded a model developed in our previous work (Therrien et al. (2016). The simple mechanistic model assumes that trial-to-trial variability in subjects’ reach angles stems from two broad sources termed “exploration variability” and “motor noise.” The important distinction between these sources of variability is that the sensorimotor system has access to the amount of exploration on any trial, but it does not have access to the motor noise on that trial. Although the model is framed in terms of motor noise and exploration variability, it is equally valid to view the motor noise as proprioceptive noise (or a combination of both motor and sensory noise), so that this noise limits the ability to localize the limb. As a result, when a reach is reinforced, the motor system can only learn from the magnitude of exploration that contributed to it. Thus, high motor noise may decrease the efficiency of learning by altering the mapping of the reach angle to the reinforcement signal. Here, we allowed exploration to vary depending on whether the previous trial was rewarded or not. Fitting the model to an individual participant’s task performance revealed that added noise increased the fitted motor noise in healthy participants to match that found in patients, but there were group differences in exploration variability. While patients with cerebellar damage showed similar exploration following rewarded trials compared with healthy control subjects with and without added noise, their exploration following unrewarded trials was reduced. This suggests that the patient group was less able to modify their behavior following errors than healthy participants, even when the level of noise was matched between groups.

A discrepancy in error sensitivity between our high-noise condition and the patients with cerebellar damage could have arisen for a number of reasons. Studies of visuomotor adaptation have shown that healthy individuals are able to detect false or variable feedback and explicitly alter their behavior so as to learn normally (Bond and Taylor, 2017; Morehead et al., 2017). Added noise in the present study was akin to providing participants with false feedback. Given that they had normal proprioceptive precision, it is possible they were aware of a discrepancy between the movements performed and the feedback received, which may have reduced their sense of agency over feedback about performance errors (Parvin et al., 2018). Furthermore, healthy participants may have been able to use an estimate of the discrepancy to adjust their response to achieve more rewarding feedback. In contrast, pathological motor variability from cerebellar damage is considered to be the product of faulty predictions of limb states (Miall et al., 2007; Miall and King, 2008), which result in poor compensation for limb dynamics and interjoint interaction torques during movement (Bastian et al., 1996; Bhanpuri et al., 2014). Therefore, in patients with cerebellar damage, noise may increase uncertainty about the movement performed—that is, decrease proprioceptive precision (Bhanpuri et al., 2013; Weeks et al., 2017a,b). While the feedback resulting from such movements can also be viewed as false, patients with cerebellar damage are likely to be less able to detect and estimate the discrepancy, making it difficult to detect the source of errors.

Previous work has addressed how motor noise can alter learning in a variety of motor tasks. There are several studies of error-based learning that have artificially added noise into various sensorimotor tasks. These have shown that, although performance degrades, participants change their behavior so as to be close to optimal in performance given the noise (Baddeley et al., 2003; Trommershäuser et al., 2005; Chu et al., 2013). Our finding that motor noise can impair motor learning is in agreement with a recent study of reinforcement learning by Chen et al. (2017). The purpose of that study was to understand the similarities between motor reinforcement and decision-making using tasks that were designed to have similar structures. They found that the decision-making task was learned faster and suspected that this was due to the motor noise present in the motor reinforcement task. In a separate experiment, they measured the level of motor noise outside of the reinforcement learning task and showed that the level of noise was inversely related to learning. That is, participants with more noise learned slower. However, they were able to equilibrate performance by artificially adding noise into the decision-making task. This suggested, as in our experiment, that variability from noise limits the ability to learn from reinforcement feedback.

In conclusion, we have shown that adding external noise to the movements of neurologically healthy individuals alters reinforcement learning in a motor task. Our findings suggest that high levels of noise primarily impair learning through the attribution of reinforcement to incorrect behavior. Not reinforcing correct behavior did not impair learning in our task, suggesting that it is less detrimental to the motor system. Additionally, adding noise to healthy individuals’ reaches reduced total learning to a level similar to that of a group of patients with cerebellar damage. However, healthy participants showed a faster initial learning rate. We suggest that this may result from a discrepancy between the form of noise in the present study and the source of noise in the patients with cerebellar damage. That is, the added noise in our experiment did not disrupt participants’ estimate of their actual behavior. This left a sufficient proportion variability accessible to the sensorimotor system, which may have supported a faster learning rate.

## Synthesis

Reviewing Editor: Robert Kalb, Northwestern University Decisions are customarily a result of the Reviewing Editor and the peer reviewers coming together and discussing their recommendations until a consensus is reached. When revisions are invited, a fact-based synthesis statement explaining their decision and outlining what is needed to prepare a revision will be listed below. The following reviewer(s) agreed to reveal their identity: R. Christopher Miall, Joseph Galea.

Both reviewers are enthusiastic about the work but both would like changes to the manuscript. Both reviewers ask explicitly for more information about the model and reviewer #2 in particular would like some of the data presented in a different way. The reviewer's specific comments are appended below:

Rev 1

This paper is a nice extension of the paper by Therrien and colleagues (2016), and reuses some of the data from that study on cerebellar patients to compare to new data reported here on healthy participants. In either participant group, the subjects made reaching movements to a visual target and received reinforcement if they reached with sufficient accuracy. The 2016 report had suggested that the patients' performance was limited by poor knowledge of the movement outcomes, due - they proposed - to added noise in the sensorimotor or predictive systems that would allow an estimate of the movement outcome, despite the lack of visual feedback. In the new paper, the authors artificially increase the noise by adding one of two levels of random noise to the final reach positions, before calculating the movement accuracy. Thus, reinforcement on some trials may incorrectly reward an inaccurate movement or fail to reward an accurate one.

They show convincing data that this reduces learning, and modelling based on the same simple model used by Therrien et al. confirms that the noise in feedback is equivalent to added motor noise, but not exploration noise, and degrades learning in an analogous fashion to the patients.

The only issues in the current paper are that it is difficult to understand the model fitting process, , and even with reference to the earlier paper the description is limited.

In addition, the results suggest that the participants differ importantly in how they treat “false hits” - trials in which the movement is inaccurate but reward is delivered, compared to “false misses”. The model would be corrupted by the false hits, and is unchanged by false misses, but is agnostic to the size of the mismatch. In contrast, the participants might be aware of some of the more extreme false feedback trials (e.g. when the reward was delivered for an end position noticeably different to the target location). Given a small reward zone, participant may be less able to detect a false miss trial (where the error between their perceived position and the target zone could be small) than a false hit trial, where the perceived distance could be large enough to be detected. As only hits (true or false) update the model, then there is an asymmetry here; it would be good to discuss this issue in more detail. It might be worth examining the distribution of rewarded end-position errors in Expt 1 and 2. It would be great if the authors would test their prediction (line 381) that the false hits are important with an additional condition.

Additionally, the authors might want to cite the commentary on their original paper, where this experimental manipulation of feedback was suggested, among others.

As a minor point, the reward clamping procedure in Experiment 2.1 and 2.2 might lead to a lower reinforcement rate towards the later stages of the experiment, as the clamp attempts to reduce the mean reward rate. This should be tested.

The bar plots in Figure 3C, 5D-F and 6A-C should be labelled, rather than only relying on color.

Rev 2

The current manuscript investigates the role of motor variability in reinforcement motor learning. Across several experiments the authors show that increased motor noise slows reinforcement learning by distorting the mapping between reward and successful actions. A link is made between artificial noise in healthy volunteers and increased motor noise in cerebellar patients. This is a well written, clear and interesting study with generally sound results. However, I do believe there are several aspects which must be addressed:

Group sizes: The authors should explicitly state in the methods their choice of group sizes. If no power analysis was performed then this should be stated.

Figure 2: Why did the authors choose to show a participant that doesn't really show any learning (i.e. their reach angle does not deviate away from 0 during the visuomotor displacement blocks, apart from maybe 1-2 trials)?

Figures 3-6: I believe the manuscript would benefit from figures which show individual data rather than simply bar graphs representing mean +- SEM (figures 3-6). Could these bar graphs be overlaid with individual data points from each participant? Alternatively the authors could use box plots or some other form of distribution plots. Following the data shown in figure 2, I am intrigued to know how many participants successfully adapted their reach angle in the control sessions.

Bonferroni corrections: Could more information be provided regarding these corrections. For example, what correction was applied for the results provided between lines 304-317?

Model results: The model results require further justification (it is not sufficient to say this is covered in the Therrien et al. paper). (i) The authors provide no evidence of how well this model fitted each participant's data. What was the R-square range? (ii) Were the 2 parameters of the model correlated? If so, this becomes a problem. (iii) Could the authors also provide partial R-square results (setting one parameter to zero and rerunning the model) for each model parameter? This will reveal the importance of each parameter to the model (assuming they are independent). The authors could then use Akaike's information criterion (AIC) or Bayesian information criterion (BIC) for model comparisons.

Planned comparisons: The planned t-tests with the model (lines 435 onwards) require further justification. Why are these planned? Did the authors pre-register these planned comparisons somewhere? I am assuming the “planned” nature of these t-tests made them 1-tailed and not corrected for multiple comparisons? If so, this would make the p=0.011 on very rocky ground. Also the t-test reported on line 450, is this corrected for multiple comparisons?

Angular changes following success/failure: I find the results difficult to comprehend as performance has simply been averaged across blocks. I have always found the analysis performed by Pekny et al., (2015) to be a great way to decompose reward-based learning. Could the authors investigate trial-by-trial behaviour in a manner similar to Pekny et al., (2015)? Specifically, comparing change in reach angle following a rewarded or unrewarded trial. This could be further decomposed into change in reach angle following reward-correct, reward-incorrect, unrewarded-correct, unrewarded-incorrect trials.

Pekny 2015 results: Following this, how do the authors integrate their results from experiment 2 with the Pekny results? Specifically, Pekny et al., (2015) showed that altering the probability of reward following a successful trial from 100% to 40% significantly increased reaching variability/exploration. Based on these results, I would have predicted that the clamp-46 & 34 groups would at the very least show greater variability/exploration. If the authors apply their model to these control participants, do they observe greater exploration?
